# MISC: missing imputation for single-cell RNA sequencing data

**DOI:** 10.1186/s12918-018-0638-y

**Published:** 2018-12-14

**Authors:** Mary Qu Yang, Sherman M. Weissman, William Yang, Jialing Zhang, Allon Canaann, Renchu Guan

**Affiliations:** 1 Joint Bioinformatics Program, University of Arkansas Little Rock George Washington Donaghey College of Engineering & IT and University of Arkansas for Medical Sciences, Little Rock, AR 72204 USA; 2 Department of Genetics, Yale University, New Haven, CT 06512 USA; 3 Department of Computer Science, Carnegie Mellon University School of Computer Science, Pittsburgh, PA 15213 USA; 4 College of Computer Science and Technology, Jilin University, Changchun, Jilin 130012 China

**Keywords:** Missing data, Single-cell RNA-seq, False negative curve, Zero-inflated model

## Abstract

**Background:**

Single-cell RNA sequencing (scRNA-seq) technology provides an effective way to study cell heterogeneity. However, due to the low capture efficiency and stochastic gene expression, scRNA-seq data often contains a high percentage of missing values. It has been showed that the missing rate can reach approximately 30% even after noise reduction. To accurately recover missing values in scRNA-seq data, we need to know where the missing data is; how much data is missing; and what are the values of these data.

**Methods:**

To solve these three problems, we propose a novel model with a hybrid machine learning method, namely, missing imputation for single-cell RNA-seq (MISC). To solve the first problem, we transformed it to a binary classification problem on the RNA-seq expression matrix. Then, for the second problem, we searched for the intersection of the classification results, zero-inflated model and false negative model results. Finally, we used the regression model to recover the data in the missing elements.

**Results:**

We compared the raw data without imputation, the mean-smooth neighbor cell trajectory, MISC on chronic myeloid leukemia data (CML), the primary somatosensory cortex and the hippocampal CA1 region of mouse brain cells. On the CML data, MISC discovered a trajectory branch from the CP-CML to the BC-CML, which provides direct evidence of evolution from CP to BC stem cells. On the mouse brain data, MISC clearly divides the pyramidal CA1 into different branches, and it is direct evidence of pyramidal CA1 in the subpopulations. In the meantime, with MISC, the oligodendrocyte cells became an independent group with an apparent boundary.

**Conclusions:**

Our results showed that the MISC model improved the cell type classification and could be instrumental to study cellular heterogeneity. Overall, MISC is a robust missing data imputation model for single-cell RNA-seq data.

## Background

Single cell genomic analysis has made it possible to understand cellular heterogeneity [1]. Advances in single cell genomics research have also provided unprecedented opportunities in biomedical research where it is important to identify different cell types pertinent to aging and cellular malignancy. Currently, completely eliminating cancer using molecularly targeted therapies is still a distant goal for many types of malignancy. Thus, investigating rare cancer stem cells that are resistant to therapy and studying intratumoral heterogeneity with differential drug responses in distinct cell subpopulations provides a basis for approaching this goal [2]. Over the past 5 years, single cell studies that aimed at the scale and precision of the genome-wide profiling of DNA [3], RNA [4], protein [5], epigenetics [6], chromatin accessibility [7], and other molecular events [8] have reached tens of thousands of cells for massively parallel single-cell RNA sequencing [9] and millions of cells for mass cytometry signature protein measurements [10]. Newer and better methods for conducting single cell analyses can capture cell population heterogeneity, including cancer’s heterogeneous nature, and facilitate the discovery of the underlying molecular mechanisms.

Although single-cell RNA sequencing (scRNA-seq) data analysis provides us an opportunity to study the heterogeneity of cells and the genes that are differentially expressed across biological conditions, it is a challenging process to perform the analysis. With the fast-increase in scRNA-seq data, computational methods need to overcome challenges ranging from handling technical noise to constructing and characterizing cell identities, and to cell lineage analysis through computing high-dimensional sparse matrixes. Therefore, innovative, efficient, robust, and scalable computational analysis methods are essential to this new frontier.

Currently, the main obstacle in scRNA-seq data analysis, stems from low capture efficiency and stochastic gene expression, which increases gene dropout events in genome-wide scRNA-seq data. We designate these dropout events as the missing data events of single-cell data. Previous studies indicate that the overall missing rates are consistently high in some single-cell data. For example, in a mouse embryo cell, the missing rate can reach nearly 30%, even after noise reduction [11] With a high fraction of missing data, direct deletion of the missing data can result in a loss of valuable information [12]. To yield better separation of different cell types and reveal new biologically meaningful subpopulations, several publications have reported the missing data as censored data and false negative error [13–15]. All these methodologies assume the distribution of the missing data; however, deriving adequate probability distributions is a difficult problem [12]. In 2016, Regev et al. noted that missing data (false negatives), false positives, and data sparsity can strongly affect the estimates of cell heterogeneity, thus new methods as well as the effectively adaption of existing algorithms are required [1]. Additionally, traditional missing data imputation, such as user-based and item-based joint filtering, often assumes that the missing positions are already known in the matrix [16]. Nevertheless, there are still key questions about scRNA-seq expression matrices that need to be addressed. Without the missing position information, the aforementioned data imputation methods cannot be utilized.

To solve the key problems in missing value imputation, we proposed a novel model with a data-driven machine learning method, namely, missing imputation on single-cell RNA-seq (MISC). The MISC was designed to address three problems: where the missing data is?; how many pieces of data are missing?; and what their values are?. Its initiation involves modeling the problem to transform the missing data imputation into two machine learning problems for detection and imputation of the missing data events. Then, we proposed a model based on classification and regression methods to solve the aforementioned problems. Finally, we evaluated the missing imputation method on two real datasets for studies of differentiating cells and cell - type detection.

## Methods

**Fig. 1:**
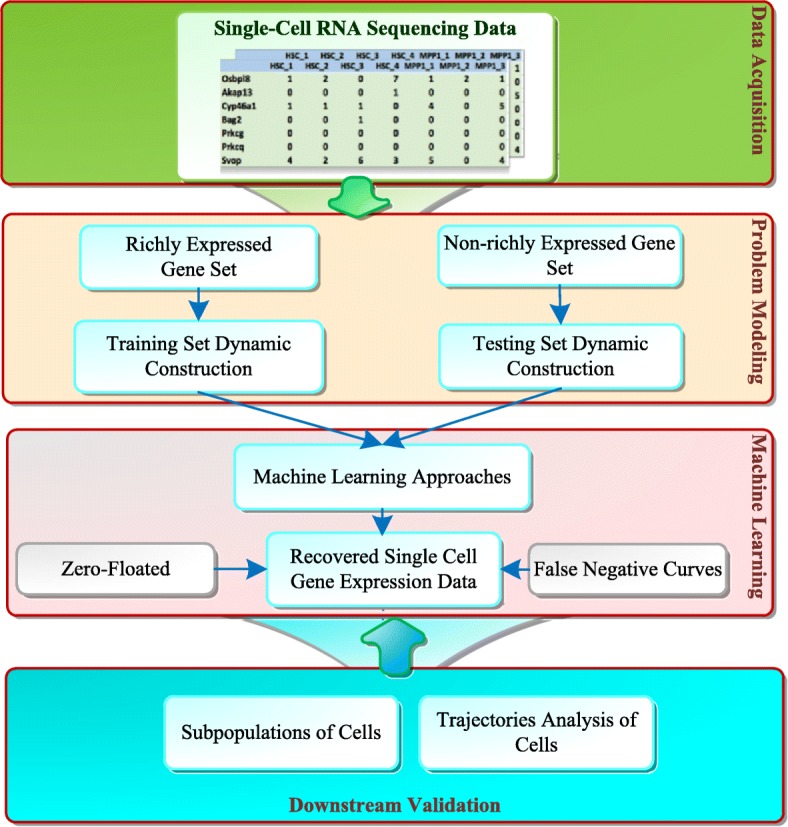
Flowchart of missing imputations on single-cell RNA-seq (MISC). It consists of data acquisition, problem modeling, machine learning and downstream validation. The machine learning approach includes binary classification, ensemble learning and regression

There are four modules (data acquisition, problem modeling, machine learning approach and downstream validation) in our scRNA-seq missing data discovery flowchart (Fig. 1). First, the scRNA-seq genome-wide data are collected. In our experiments, we collected datasets from stem cells of chronic myeloid leukemia [2] from mouse brain cortex and the hippocampus [17], respectively. Then, using problem modeling and machine learning approaches, the RNA-seq expression of the missing data can be detected and recovered. For the first problem, where data is missing, we transformed this problem into a binary classification on the RNA-seq expression matrix in which each element represented a sample. Then, for the second problem, how many data points are missing, we searched for the intersection of the classification results, between the zero-inflated model (ZIM) and the false negative model (FNC) results. Because the latter two models are not mainly focused on the missing data problem (one is for the identification of the subpopulations of cells, and the other is for the visualization of the single-cell data), they only provide the probability matrixes of the missing data. We selected the top missing elements in the matrixes with a threshold *η*. In which, *η* can be computed using the rate of classification results and the counts of the test dataset. Finally, to determine their values, we used a regression model to impute the data in the missing elements.

In the second module, the problem modeling, single-cell missing data was first transformed into a binary classification set. The hypothesis is: if the classifier finds a group of richly expressed genes, whose expression values are equal to zero, than these expressions should be non-zeros and missing values. For the different data, the richly expressed genes can be projected on different gene sets from other genomics data. We used the expression values of these genes as a training set to guide the binary classification model and detect the missing elements in the whole RNA-seq matrix. First, to pursue the latent patterns of the missing data, we constructed a training set based on the matrix transformation of richly expressed genes. All the genes are split into richly expressed gene sets and non-richly expressed gene sets. With these two gene sets, we can construct the richly expressed gene expression matrix as training data and the non-richly expressed gene expression matrix as test data. The positive set is all the gene expression values larger than zero in a single-cell RNA-seq expression matrix and the negative set is all the values equal to zero.

Suppose an element *x*[*i*, *j*] in which *X* indicates the expression matrix of the richly expressed genes, 0 < *i < m*, 0 < *j* < *n*, where *m* indicates the number of genes, and *n* is the number of cells. In generated training set, each element *x*[*i*, *j*] is a sample and the its features *j*’ are *j*’ ≠ *j*, 0 < *j*’ < *n*. The missing data value *y*_*i,j*_ of a typical gene *j* in one cell *i* can be predicted with the gene expression values.

$$ {Y}_{i,j}=\mathit{\operatorname{sgn}}\left(F\left(x\left[i,{j}^{'}\right]\right)\right),{j}^{'{}^1}j,0<{j}^{'}<n $$

where sgn(•) is the sign function, and *F* is the machine learning function. Therefore, the training set *s* has *m* × *n* samples, and the feature set *f* contains *n*-1 features. In our case, we took the mouse cortex and hippocampus data as an example for the process. The training set has 406 genes (m), 3,005 cells (n), 1,220,030 samples (m x *n* = 406 × 3005) and 3,004 features. Similarly, the test set contains *t* × *n* samples and *t* is the number of non-richly expressed genes. In the example, the test set has 19,566 genes (m), 3,005 cells (n), 58,795,830 samples and 3,004 features.

In the third module, with the aforementioned problem modeling, it can be seen that the computational complexity reaches *O*(*mn*^2^). Considering the fast development of the single cell experiments, which can perform up to tens of thousands of single cells [1], we employed a large linear classification (LLC) *F* to discover the missing data, which is of much efficiency for the large data set. The method involves solving the following optimization problem:

$$ \underset{w}{\min}\frac{1}{2}{w}^Tw+C\sum \limits_{i=1}^l\xi \left(w,{s}_i,{y}_i\right), $$

where *s* is the sample, *y* is the class label for the classification and the expression value for regression, *w* is the weight vector and *w*∈*R*^*n*^, *C* is the penalty factor, *C* > 0. We adopted two popular binary linear classifiers, named Logistic Regression (LR) and a Support Vector Machine (SVM) with a linear kernel. LR with L2-regularization employs the following unconstrained optimization function.

$$ \underset{w}{\min}\frac{1}{2}{w}^Tw+C\sum \limits_{i=1}^l\log \left(1+{e}^{-{y}_i{w}^T{s}_i}\right). $$

The correspondence dual form is

$$ \underset{\alpha }{\min}\frac{1}{2}{\alpha}^T Q\alpha +\sum \limits_{i:{a}_i>0}^l{a}_i\log {a}_i+\sum \limits_{i:{a}_i<C}\left(C-{\alpha}_i\right)\log \left(C-{\alpha}_i\right),\mathrm{Subject}\ \mathrm{to}\ 0\le {\alpha}_i\le C,i=1,\dots, l. $$

Then, the problem can be solved with a trust region Newton method [18] or dual coordinate descent method [19] SVM with L2-regularization on L2-loss uses the following unconstrained optimization function

$$ \underset{w}{\min}\frac{1}{2}{w}^Tw+C\sum \limits_{i=1}^l\log {\left(\max \left(0,1-{y}_i{w}^T{s}_i\right)\right)}^2. $$

The dual form is

$$ \underset{\alpha }{\min}\frac{1}{2}{\alpha}^T Q\alpha -{e}^T\alpha, {Q}_{ij}={y}_i{y}_j{s}_i^T{s}_j,\mathrm{Subject}\ \mathrm{to}\ 0\le \alpha \le U,i=1,\dots, l. $$

Then, the problem can be solved with a coordinate descent algorithm [20].

To further validate the missing data and their percentage, we employed our linear classification model, the zero-inflated model [14] and false-negative curves [15] to construct an ensemble learning method. The zero-inflated model was used as a mixture model for read counts in which the first one is a negative binomial (NB) and the second is a low-magnitude Poisson. For example, given a single cell *c*, the reads *r*_*c*_ were modeled as a mixture of “drop-out” data with Poisson (*λ*_*0*_) and “amplified” components with *NB*(*e*), where *e* is the expected expression magnitude, and the background read frequency for dropout was *λ*_*0*_ = 0.1. To fit the mixture model, a subset of genes should be selected. First, given a subpopulation of cells, all the pairs of individual cells (*r*_*i*_, *r*_*j*_) were analyzed with the following model.

$$ \left\{\begin{array}{ccc}{r}_i\sim P\left({\lambda}_0\right)& dropout& in\kern0.5em {c}_i\\ {}\left\{\begin{array}{c}{r}_i\sim NB\left({r}_j\right)\\ {}{r}_j\sim NB\left({r}_i\right)\end{array}\right.& amplified& \\ {}{r}_j\sim P\left({\lambda}_0\right)& dropout& in\kern0.5em {c}_j\end{array}\right., $$

Then, a multinomial logistic regression (the mixing parameter *m* = log(*r*_*i*_) + log(*r*_*j*_)) and an expectation–maximization algorithm was used to fit the model. The genes that were assigned to the “amplified” components could be noted, and the set of genes appearing in the amplified components in at least 20% of all the comparisons of the same subpopulation of cells were used to fit the model.

False-negative curves employ housekeeping genes to fit a logistic regression function *F*_*c*_(*μ*) whose odds quantify the cell’s technical detection efficiency [1] In a given gene, its expected expression μ* is conditioned to be detected and 1- *F*_*c*_(μ*) is the missing probability of this gene in cell *c*.

The differences among the three methods for missing data detection are the training set (subset of genes) and training (fitting) method. First, all three methods need a subset of genes to train or fit the model. From the biology view, the false negative model and large linear classification use the richly expressed genes. However, from the statistical view, the zero-inflated model uses a mixture model of Poisson and negative binomial (NB) to select a subset of genes. Moreover, both the zero-inflated model and false negative model employ logistic regression to fit a model for each cell RNA-seq expression value. The large linear classification uses a linear model instead of a logistic function, which is efficient for big data. Therefore, all three methods try to detect the missing data from different views, which satisfied the heterogenous rule of ensemble learning.

After obtaining the ensemble learning and obtaining the missing positions in the RNA-seq expression matrix, we employed a linear regression model to recover the missing values. In our experiments, we employed the support vector regression (SVR) model with a linear kernel. The training set is the same as the classification task; however, the label of the training samples using normalized RNA-seq expression values, such as reads per kilobase per million (RPKM). For the regression optimization function, we employed three L2-regularized methods, which are the dual problem solutions of L1-loss support vector regression, the primal problem solution and the dual problem solution of the L2-loss support vector regression. The L2-regularized SVR is modeled using the following optimization problems:

$$ \underset{w}{\min}\frac{1}{2}{w}^Tw+C\sum \limits_{i=1}^l\log {\left(\max \left(0,|{y}_i\hbox{-} {w}^T{x}_i|-\varepsilon \right)\right)}^p, $$

where *p* = 1 indicates the L1 loss and *p* = 2 is the L2 loss, and *ε* ≥ 0 is the sensitiveness of the loss. The dual forms of the problem are:

$$ \underset{\alpha^{+},{\alpha}^{-}}{\min}\frac{1}{2}\left[{\alpha}^{+}{a}^{-}\right]\left[\begin{array}{cc}{Q}^{\prime }& -Q\\ {}-Q& {Q}^{\prime}\end{array}\right]\left[\begin{array}{c}{\alpha}^{+}\\ {}{\alpha}^{-}\end{array}\right]-{y}^T\left({\alpha}^{+}-{a}^{-}\right)+\varepsilon {e}^T\left({\alpha}^{+}+{a}^{-}\right) $$

where *e* is the vector of all ones, *Q*’ = *Q* + *D*, *Q*_*ij*_ = *x*_*i*_^*T*^*x*_*j*_, *D* is the diagonal matrix and *p* = 1, *D*_*ii*_ = 0; *p* = 2, *D*_*ii*_ = 1/2*C*; 0 ≤ *α*_*i*_^*+*^*,α*_*i*_^*+*^ *≤ U*, *i* = 1,…,*l*, *U*=*C* when *p* = 1; *U* = ∞, and when *p* = 2. We use LIBLINEAR tool to solve this problem [20].

In addition, based on the classification results (which show the missing positions in RNA-seq expression matrix), a mean-smooth curve with the neighbor cell method on the cell trajectories is also proposed to make a comparison with the MISC. This method recovers the missing values with the expressions of the *γ* of the previous and following cells (*γ* = 3 in our experiments).

For the fourth module, we employed the trajectory analysis and subpopulation analysis to directly show the effectiveness of our MISC method.

Two real scRNA-seq datasets were used to verify the effectiveness of our model. One is chronic myeloid leukemia (CML) data (Gene Expression Omnibus: GSE76312) [2]. It is used to reveal the heterogeneity of CML stem cells and the identification of subclasses of CML stem cells. It includes five types of stem cells from either patients or normal donors, which are analyzed at different stages of the disease. The other one is the genome-wide single-cell RNA-seq data of the primary somatosensory cortex and the hippocampal CA1 region of mouse brain cells in [17] (Gene Expression Omnibus: GSE60361). It includes 3,005 single cell transcriptomes (19,972 genes) and each RNA molecule was counted using a unique molecular identifier (UMIs) (essentially tags that identify individual molecules) and confirmed by single-molecule RNA fluorescence in situ hybridization (FISH).

## Results

The CML data includes 2,287 stem cells throughout the disease course and 23,384 genes. To analyze the heterogeneity of the stem cells from normal HSCs, we selected 1,102 stem cells without tyrosine kinase inhibitor treatments. Then, the *t*-SNE analysis of these samples was performed using the top 234 differentially expressed genes with a false-discovery rate (FDR) cutoff of 0.05 and an absolute log fold change cutoff of 1. The training dataset of our MISC machine learning model is based on the richly expressed gene set, which employs human housekeeping genes from reference [21] for CML stem cell data. It contained 38 genes, 1,102 stem cells, and 41,876 samples. The corresponding test dataset includes 196 genes, 1,102 stem cells and 215,992 samples. For the large linear classifiers, we used 5-fold cross validation on the training set and achieved a classification accuracy of 0.80. Finally, for the two L2-regularization based LLCs, we selected an L2-loss support vector machine (with parameter *C* = 2) due to better accuracy. The missing rate threshold *η* = 0.35 for the false negative curve (the raw reads count data is not provided, therefore, we only use FNC method to determine the intersection). The final missing rate of CML data (the overlap of the missing data sets between MISC and FNC method) is 13.6%. After several parameter selection experiments, we selected L2-loss support vector regression with primal problem solution (parameter *C* = 0.125) due to its lowest mean-square error among the three regression methods.

**Fig. 2:**
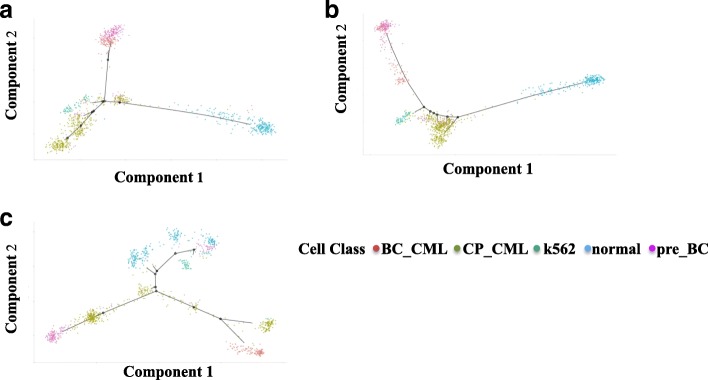
Missing data imputation benefits to reveal CML stem cell trajectories associated with disease progression in CML. The trajectories include five types of stem cells, CP-CML in black (*n* = 477), normal HSCs in blue (*n* = 232), pre-BC samples taken as the patients presented in CP (pre-BC) 12 months and 3 months before transformation to myeloid and lymphoid BC in green (*n* = 185), BC-CML in purple (*n* = 155) and K562 in red (*n* = 53) using the top 234 differentially expressed genes. **a** The single-cell RNA-seq expression trajectories analyzed on CML stem cells without data imputation. **b** The trajectory analysis on CML stem cells using the mean-smooth method with neighbor cells on the trajectory. **c** The trajectory analysis on CML stem cells using MISC methods to recover the CML data

For single-cell trajectory analysis, five different types of stem cell chronic-phase CMLs (CP-CML), normal hematopoietic stem cells (HSCs), pre-BC samples taken from the patients who were presented in CP (pre-BC) 12 months and 3 months before transformation to myeloid and lymphoid blast crisis (BC), blast crisis CML (BC-CML), K562 human erythroleukemic cell lines derived from a patient in CML blast crisis appear in branches in trajectories during cell development in Fig. 2. Using the top 234 differentially expressed genes, 1102 stem cells without any imputation methods (Fig. 2a) show the branches of CP-CML but failed to divide the pre-BC and BC-CML cells. The mean-smooth neighbor cells on the trajectory method (Fig. 2b) strips the BC-CML from the pre-BC cells; however, the branches of CP-CML have been weakened. The MISC method (Fig. 2c) clearly divides the BC-CML and pre-BC cells. Furthermore, the RNA-seq expression data shows a trajectory branch from CP-CML to BC-CML, which provides direct evidence of the evolution from CP to BC stem cells. In reference [2], a similar result was achieved by clustering, which consists of both of CP and BC stem cells. In addition, normal HSCs are also divided into three branches, which provide further analysis potential. One of them shows a branch mix with normal and pre-BC stem cells, which can provide clinical research opportunity.

**Fig. 3:**
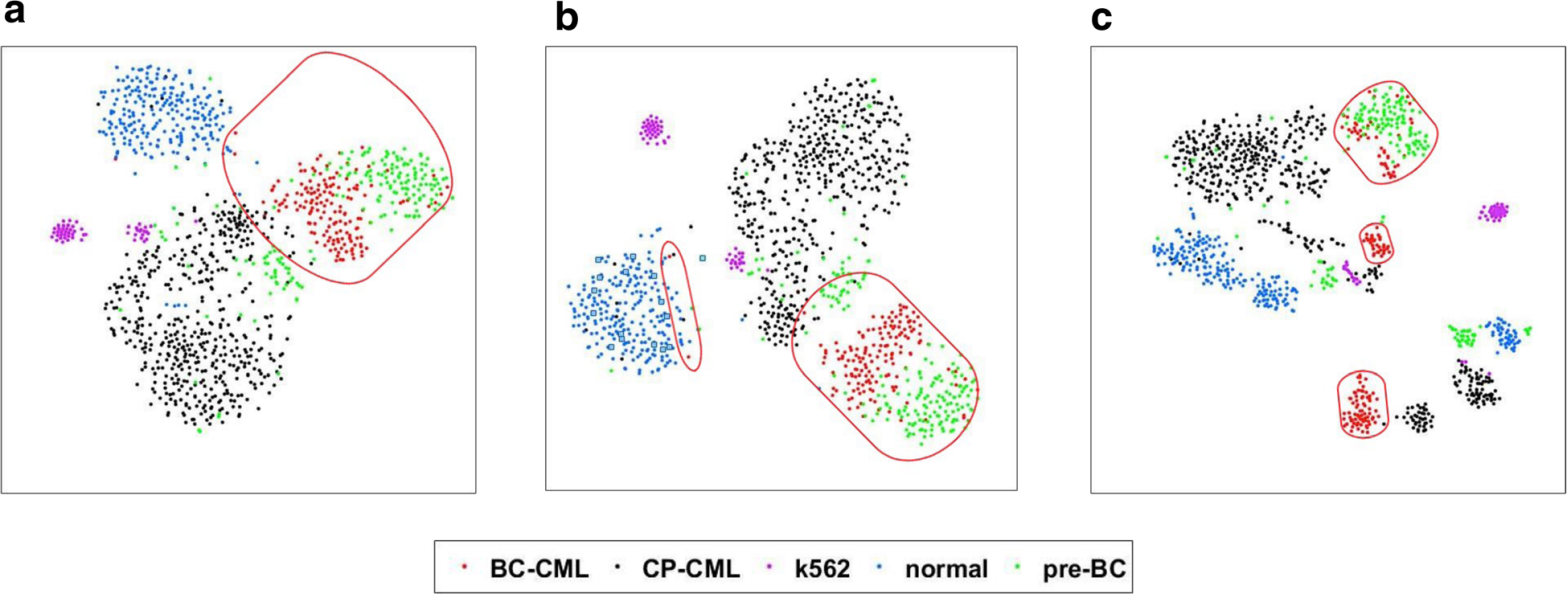
*t*-SNE analysis on imputed single-cell RNA-seq reveals more clearly subpopulations of CML stem cells. All types of these stem cells are of CP-CML in black (*n* = 477), normal HSCs in blue (*n* = 232), pre-BC samples taken from the patients presented in CP (pre-BC), 12 months and 3 months before transformation to myeloid and lymphoid BC in green (*n* = 185), BC-CML in purple (*n* = 155) and K562 in red (*n* = 53). Red ovals focus on the group of BC-CML stem cells. **a** The *t*-SNE analysis on the CML stem cell data without missing the imputation. **b** The *t*-SNE analysis on the CML stem cell data using the mean-smooth method with neighbor cells on the trajectory. **c** The *t*-SNE analysis on CML stem cell data using the MISC method

With *t*-SNE analysis, all five different types of stem cells are visualized in Fig. 3. The original distribution of the five cell types is a mess (Fig. 3a), especially for the BC-CML type in the red oval. Moreover, the CP-CML cells mix with the pre-BC cells, normal cells and K562 cells. With the mean-smooth method with neighbor cells on the trajectory, the split groups in Fig. 3b are clearer than those without missing imputation. However, there are two cells are mixed with normal HSCs. The *t*-SNE visualization on the single-cell RNA-seq data using MISC imputation (Fig. 3c) shows the clearest groups among the three figures. Furthermore, the lowest red oval also proves the evolution from CP to BC stem cells as our trajectory analysis. In addition, the MISC imputed single-cell RNA-seq data present more compact clusters in Fig. 3c, which provides opportunities for subpopulations and rare cell type analysis on CML stem cells. From Figs. 2 and 3, it can be seen that the MISC data imputation method can help to analyze the trajectory branches of CML stem cells and their subpopulation detection.

**Fig. 4:**
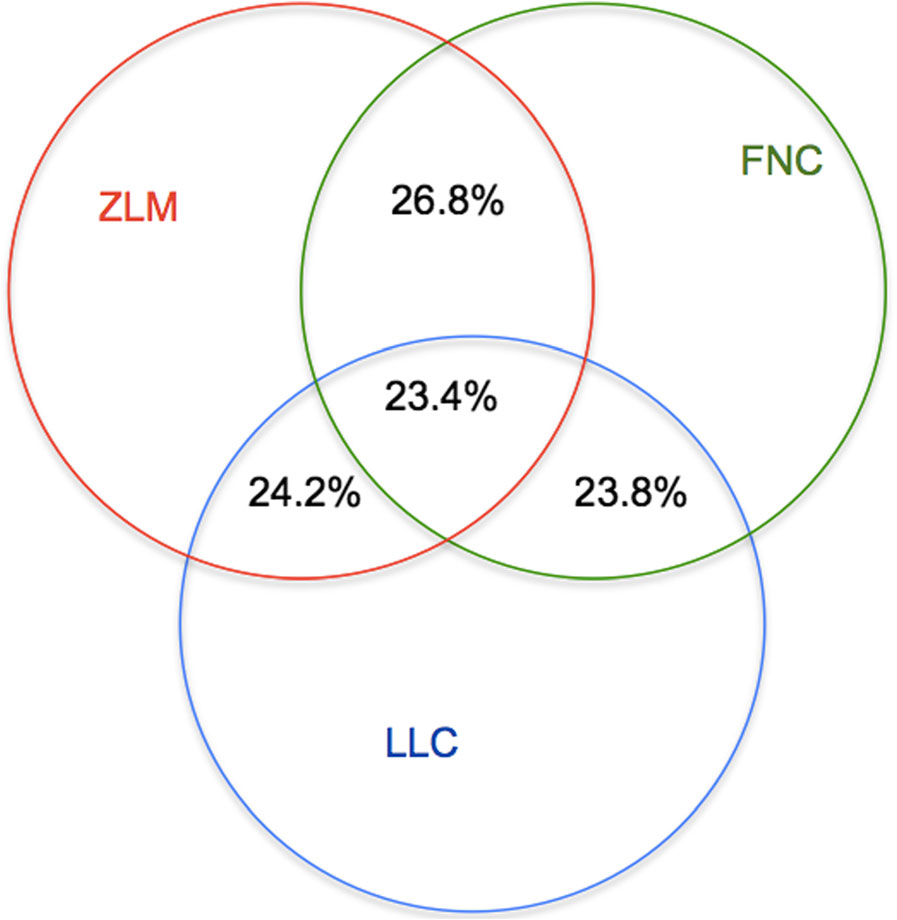
The overlap of the missing data discovered by ZIM, FNC and LLC. The red circle is the missing data discovered by the zero-inflated model (ZIM); the green circle is false negative curve (FNC); the blue circle is from large linear classification (LLC). LLC∩ZIM = 11,117,664,47.6%; LLC∩FNC = 11,040,187, 47.2%; ZIM∩FNC = 11,745,190, 50.2%; LLC∩ZIM∩FNC = 5,493,856, 23.4%

For the primary somatosensory cortex and hippocampal CA1 region, the single cell data contains 19,972 genes, including 406 housekeeping genes (using the same list in reference [15]) and 3,005 cells. Therefore, the training set contains 1,220,030 samples and the test set, includes 58,795,830 samples. For the large linear classifier (LLC), we used 5-fold cross validation on the training set and achieved 80% accuracy as the CML data. Finally, for the two L2-regularization based LLCs, we selected the L2-loss Logistic Regression (with parameter *C* = 104.858) due to better accuracy. The missing rate threshold *η* = 0.397 for the false negative curve (FNC) and zero-inflated model (ZIM). The final missing rate of the primary somatosensory cortex and hippocampal CA1 region of mouse data is 23.4% (Fig. 4). It is approximately 10% higher than the CML data due to these data using 19, 972 genes without differential gene filters. At last, after several parameter selection experiments, we selected L2-loss support vector regression with the primal problem solution (parameter *C* = 4) due to its lowest mean-square error among the three regression methods.

**Fig. 5:**
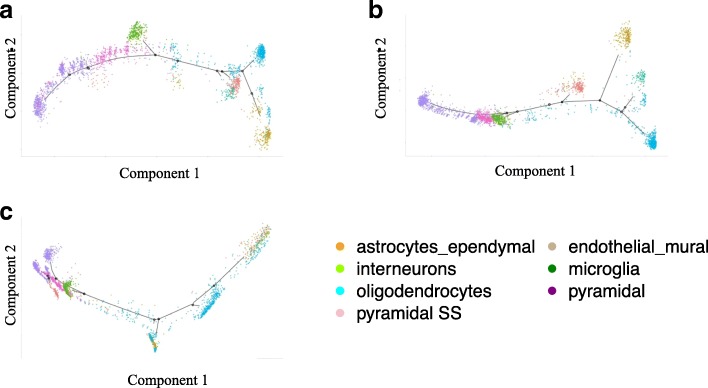
Missing data imputation benefits to recover the trajectories of the primary somatosensory cortex and the hippocampal CA1 region single-cell RNA-seq data. The trajectories include seven cell types, such as astrocytes_ependymal in orange (*n* = 224), interneurons in chartreuse (*n* = 290), oligodendrocytes in aqua (*n* = 820), pyramidal SS in pink (*n* = 399), endothelial−mural in khaki (*n* = 235), microglia in green (*n* = 98) and pyramidal CA1 in purple (*n* = 939). **a** The single-cell RNA-seq expression trajectory analysis on the mouse brain cells without data imputation. **b** The trajectory analysis on the mouse brain cells using the method of mean-smooth neighbor cells on the trajectory. **c** The trajectories analysis on the mouse brain cells using MISC method to impute CML data

For single-cell trajectory analysis, seven different types of cells, astrocytes-ependymal, interneurons, oligodendrocytes, pyramidal SS, endothelial-mural, microglia and pyramidal CA1, appeared in branches in trajectories in Fig. 5. Using all the 19,972 genes, 3,005 brain cells without any imputation methods (Fig. 5a) show the branches of astrocytes-ependymal, interneurons, oligodendrocytes, endothelial−mural and microglia, but failed to divide the pyramidal SS and pyramidal CA1 cells. The mean-smooth neighbor cells method (Fig. 2b) strips the pyramidal SS from the pyramidal CA1 cells; however, all the pyramidal CA1 in purple 939 cells stay in one branch. The MISC method (Fig. 2c) clearly divides the pyramidal CA1 into different branches, which is direct evidence that pyramidal CA1 has subpopulations [17]. Furthermore, the RNA-seq expression data shows a sub-branch at the middle left of Fig. 5a, which provides direct evidence of the subclasses of brain cells.

**Fig. 6:**
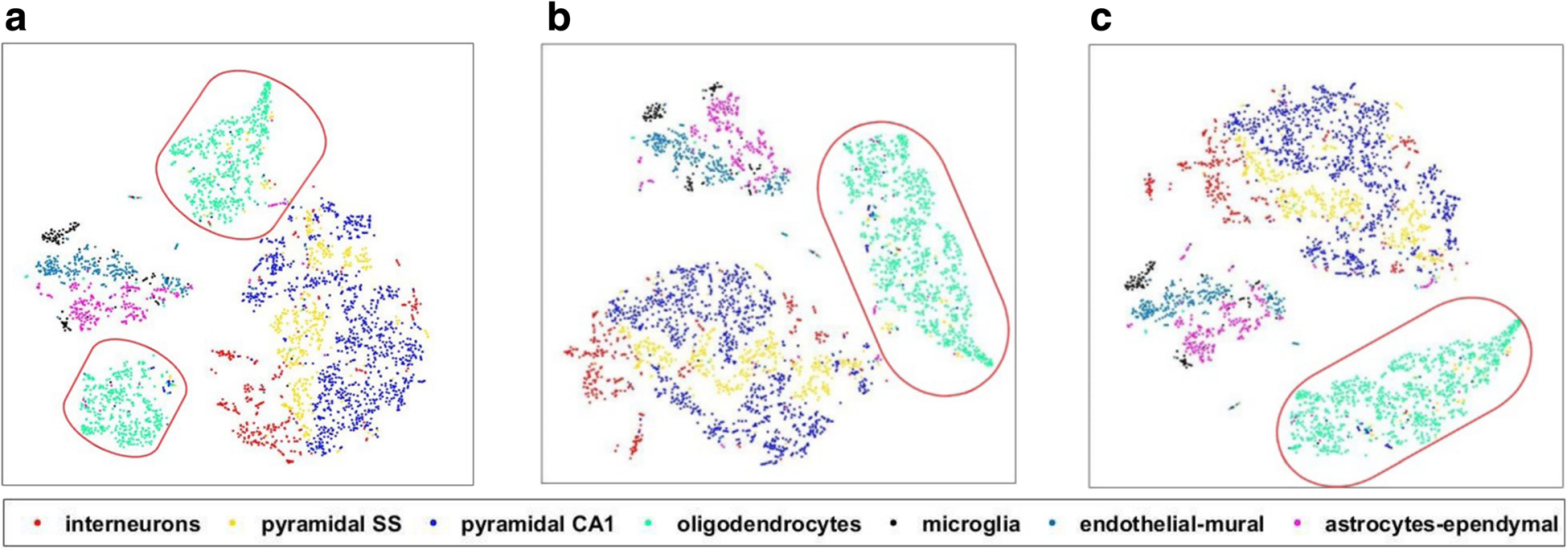
*t*-SNE analysis on imputed single-cell RNA-seq reveals cell populations of the primary somatosensory cortex and the hippocampal CA1 region of mouse brain cells. All types of these stem cells are interneurons in red (*n* = 290), pyramidal SS in yellow (*n* = 399), pyramidal CA1 in blue (*n* = 939), oligodendrocytes in cyan (*n* = 820), microglia in black (*n* = 98), endothelial-mural in teal (*n* = 235) and astrocytes-ependymal in pink (*n* = 224). Red ovals focus on the group of oligodendrocyte cells. **a** The t-SNE analysis on the mouse brain cell data without missing data imputation. **b** The t-SNE analysis on the mouse brain cell data using the mean-smooth method with neighbor cells on the trajectory. **c** The t-SNE analysis on mouse brain cell data using the MISC method

The complex brain cognitive functions, such as social behaviors and sensorimotor integration, rely on a diverse set of differentiated cells [17]. Therefore, accurate classification of the brain cell types is essential to understand the cognitive functions of the brain. Using MISC, we imputed the scRNA-seq data of the primary somatosensory cortex and the hippocampal CA1 region of the mouse brain cells. The imputation results are shown in Fig. 6. The oligodendrocyte cells in the original data without data imputation were divided into two groups (Fig. 6a). Using mean-smooth neighbor cells on trajectory imputation, these divided cells that previously were merged together (Fig. 6b); however, it can be seen that these oligodendrocyte cells connect to the other big group, which mainly constitutes interneurons, pyramidal SS, and pyramidal CA1. With MISC, the oligodendrocyte cells became an independent group and its boundary was apparent, although there are few cells in the group that still need further study. The detailed branches in Fig. 5 and the more apparent groups in Fig. 6 indicates that the MISC model can also recover the primary somatosensory cortex and the hippocampal CA1 region of mouse brain cells.

## Discussion

The dropout events are abundant in the single-cell sequencing data [13, 22]. The missing data imputation is essential for reliable downstream analysis. Most existing data imputation methods are designed to handle bulk-level data. The latent missing data distributions between single-cell and bulk-level data are very distinct. The data missing rate for scRNA-seq data is significantly higher than the one for bulk-level data. For example, the missing rate of a scRNA-seq dataset can be over 80% [17]. Additionally, the zeros in the scRNA-seq matrix either reflect the true biological values or cause by dropout. To accurately impute missing values, we developed a new method that decomposed the data imputation into three subsequent steps: missing position detection, position refinement via ensemble learning, and imputation. Our method was designed for imputing only the expression levels of the dropout genes. To achieve this, we included a refinement step to identify the missing positions with high confidence. The positions that were simultaneously detected by our model and the other two methods [14, 15] were considered as true missing positions. This strategy can improve the specificity of missing value detection. We examined the MISC model using the chronic myeloid leukemia and mouse brain scRNA-seq datasets [2, 17]. The experimental evidences suggested that our model could help to optimize the construction of cell trajectory and enable more accurate cell type detection.

The linear classification was used to achieve efficiency in computational time in our method. A more sophisticated model might provide better performance at the cost of computational expense. Hence, the method coupling parallel computing and advanced modeling could help to enhance the efficiency and accuracy of single cell data imputation. Our missing position refinement via ensemble learning may potentially exclude true missing positions. With a better model, we can also address this limitation.

## Conclusions

Single-cell RNA-seq expression profiling offers a static snapshot of the gene expression, provides estimates of cell heterogeneity and rare cell type detection. Through successfully solving the three problems of missing data, the proposed model MISC can effectively recover the missing values in the scRNA-seq data. Regarding the chronic myeloid leukemia data, MISC discovered a trajectory branch from CP-CML to BC-CML, which provides direct evidence of evolution from CP to BC stem cells. Meanwhile, *t*-SNE on MISC imputed data proves the evolution from CP to BC stem cells as our trajectory analysis and presents more compact clusters. On the primary somatosensory cortex and the hippocampal CA1 region of mouse brain cells, it clearly divides the pyramidal CA1 into different branches, it is a direct evidence of pyramidal CA1 has subpopulations. In addition through the use of MISC, oligodendrocyte cells became an independent entity with an apparent boundary. Furthermore, for filtered CML data, the MISC model can present a clear trajectory and cell type classification. For the scRNA-seq data with a large number of genes,, MISC can also help us study the cellular heterogeneity. All this indicates that MISC is a robust missing data imputation model for single-cell RNA-seq data.

## Abbreviations

CML: Chronic myeloid leukemia
FDR: False discover rate
FNC: False negative curve
HSC: Hematopoietic stem cells
LLC: Large linear classification
LR: Logistic Regression
MISC: Missing imputation on single-cell RNA-seq
NB: Negative binomial
RPKM: Reads per kilobase per million
scRNA-seq: Single-cell RNA sequencing
SVM: Support Vector Machine
SVR: Support vector regression
ZIM: Zero-inflated model
