# Correction to: MISC: missing imputation for single-cell RNA sequencing data

**DOI:** 10.1186/s12918-019-0681-3

**Published:** 2019-01-22

**Authors:** Mary Qu Yang, Sherman M. Weissman, William Yang, Jialing Zhang, Allon Canaan, Renchu Guan

**Affiliations:** 1 Joint Bioinformatics Program, University of Arkansas Little Rock George Washington Donaghey College of Engineering & IT and University of Arkansas for Medical Sciences, Little Rock, AR 72204 USA; 2 Department of Genetics, Yale University, New Haven, CT 06512 USA; 3 Department of Computer Science, Carnegie Mellon University School of Computer Science, Pittsburgh, PA 15213 USA; 4 College of Computer Science and Technology, Jilin University, Changchun, 130,012 Jilin China

**Correction to: BMC Systems Biology 2018, 12(Suppl 7):114**

**https://doi.org/10.1186/s12918-018-0638-y**

It was highlighted that the original article [1] contained a typesetting error in the last name of Allon Canaan. This was incorrectly captured as Allon Canaann in the original article.
